# New ways to boost molecular dynamics simulations

**DOI:** 10.1002/jcc.23899

**Published:** 2015-03-30

**Authors:** Elmar Krieger, Gert Vriend

**Affiliations:** ^1^ Centre for Molecular and Biomolecular Informatics Radboudumc PO Box 9101, 6500 HB Nijmegen The Netherlands

**Keywords:** multiple time‐step, LINCS constraints, pair lists, transactional memory, YASARA

## Abstract

We describe a set of algorithms that allow to simulate dihydrofolate reductase (DHFR, a common benchmark) with the AMBER all‐atom force field at 160 nanoseconds/day on a single Intel Core i7 5960X CPU (no graphics processing unit (GPU), 23,786 atoms, particle mesh Ewald (PME), 8.0 Å cutoff, correct atom masses, reproducible trajectory, CPU with 3.6 GHz, no turbo boost, 8 AVX registers). The new features include a mixed multiple time‐step algorithm (reaching 5 fs), a tuned version of LINCS to constrain bond angles, the fusion of pair list creation and force calculation, pressure coupling with a “densostat,” and exploitation of new CPU instruction sets like AVX2. The impact of Intel's new transactional memory, atomic instructions, and sloppy pair lists is also analyzed. The algorithms map well to GPUs and can automatically handle most Protein Data Bank (PDB) files including ligands. An implementation is available as part of the YASARA molecular modeling and simulation program from www.YASARA.org. © 2015 The Authors Journal of Computational Chemistry Published by Wiley Periodicals, Inc.

## Introduction

Molecular simulations with empirical force fields like AMBER,^1^ CHARMM,^2^ or OPLS^3^ are enjoying a phase of enthusiastic interest, thanks to the arrival of personal supercomputers, that is, graphics processing units (GPUs) that can accelerate science equally well as video games. As shown by AceMD^4^ and OpenMM,^5^ classical force fields are ideally suited for GPUs, because the calculations mainly require single precision floating point operations—which are the GPU's home game.

Although the slow transfer of data between CPU and GPU initially led to the development of programs that perform all computations on the GPU and let the CPU run idle, this trend seems to reverse recently. CPU and GPU are increasingly often fused on the same chip with unified memory (AMD Kaveri, Intel Iris), solving the data transfer problem. Additionally, modern CPUs contain powerful vector instructions sets (SSE, AVX), which are too valuable to be left unused. Consequently, the GROMACS team recently achieved a very high molecular dynamics (MD) performance using CPU and GPU in parallel. We, therefore, believe that MD simulations are best approached with a capable “home base” on the CPU, which can handle the countless complications in real‐life applications (like knowledge‐based force fields,^6^ X‐ray,^7^ and nuclear magnetic resonance (NMR) refinement^8^) and offloads tasks to the GPU when beneficial. In this work, we focus on this home base and describe a number of algorithms to generally improve simulation performance, and we benchmark them on a single Intel Core i7 CPU with AVX2. Most of the algorithms are equally well suited to accelerate simulations using multiple CPUs and GPUs.

Although simulation performance is usually considered less important than accuracy (which we focused on previously^6^, ^9^), only fast simulations allow an important accuracy check: whether the force field can reproduce folding and structural changes of proteins or not.^10^

## Results and Discussion

### A mixed multiple time‐step algorithm

Raising the integration time‐step to boost MD simulation speed tends to reveal its disadvantages after a few hundred picoseconds—when the simulation suddenly blows up. Such blow‐ups originate from atoms that vibrate with high speed and accidentally experience a larger than usual force, which accelerates them to the point where the distance traveled per time‐step is so large, that reliable integration is no longer possible. The vibration then becomes self‐enforcing, until atoms jump around randomly, “infecting” others and the simulation explodes. For a given force, the acceleration is inversely proportional to the atom mass, which puts hydrogens most at risk. In our experience, hydrogen bond vibrations blow up if the time‐step exceeds about 1.75 fs, hydrogen angle vibrations become critical at 2.5 fs (especially when the time‐step for nonbonded forces is larger), and heavy atom bond vibrations around 3.5 fs.

Four solutions to deal with these vibrations are commonly used: first, one can simply increase the hydrogen masses, which slows down the vibrations.^11^ Second, one can integrate the vibrations more accurately using multiple time‐steps^12^: a large time‐step for the slowly varying intermolecular forces, and a smaller time‐step for the quickly varying intramolecular forces (including the most critical bond and angle vibrations). The stability of this approach can be improved in various ways, for example, with the mollified impulse method^13^ used in NAMD.^14^ Third, one can totally remove bond vibrations by constraining the bond lengths using algorithms like LINCS^15^ or SHAKE.^16^ And finally, one can remove hydrogen angle vibrations by “virtualizing” the hydrogen atoms^11^ (i.e., by treating them as dummy atoms without mass, whose position is recalculated from the heavy atoms at each step).

**Figure 1:**
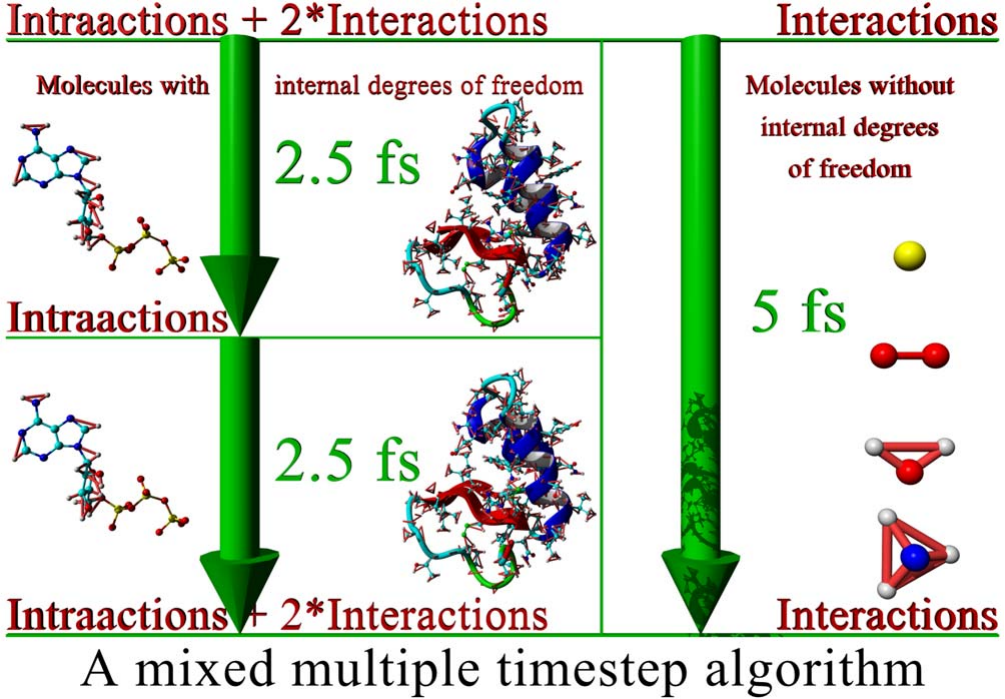
Molecular dynamics simulation by mixing a multiple time‐step algorithm (left) with a normal single time‐step algorithm (right) depending on the molecule type. The multiple time‐step algorithm uses a pulsed approach, which calculates bonded intra‐actions at each 2.5 fs step, and adds the nonbonded interactions (scaled with 2) only every other step, in sync with the 5 fs single time‐step algorithm. [Color figure can be viewed in the online issue, which is available at wileyonlinelibrary.com.]

We combine solutions two and three in a new way, which is shown in Figure 1. While a normal multiple time‐step algorithm uses the same time‐step for all atoms and a certain recipe to apply the forces (e.g., the quickly varying bonded forces at each step, and the slowly varying nonbonded forces only at every other step), we mix it with a single time‐step algorithm depending on the molecule type. Small molecules whose internal degrees of freedom can be removed by applying constraints are propagated with a large single time‐step (up to 5 fs). As all bonds and angles in such an internally frozen molecule are at their equilibrium values, the corresponding forces are zero and need not be calculated, only the nonbonded interactions are required. For all the other molecules, a multiple time‐step algorithm is used.

This approach has three advantages: first, it is easy to implement, while virtual hydrogens are rather complicated to handle (they require elaborate code for each hydrogen configuration, so that it is often not possible to simulate organic molecules “out of the box,” especially if they contain less common hydrogen configurations). Second, it does not require to change hydrogen masses (like the virtual hydrogens with zero mass or the heavy hydrogens). While it is certainly true that the effect of changing hydrogen masses is either small (compared to the errors inherent in empirical force fields) or completely absent (when looking at thermodynamic properties, which do not depend on atom masses^11^), we simply consider it convenient not having to think about the potential impact on a case‐by‐case basis. And third, it improves performance compared to normal multiple time‐step algorithms, which need to move all atoms in several costly integration steps. As shown in Figure 1, the majority of atoms (typically waters) require only a single integration step.

Care must be taken when choosing the multiple time‐step recipe because of its impact on energy conservation and simulation accuracy. In an extensive comparison study, Grubmueller and Tavan^17^ analyzed several different multiple time‐step schemes, some of which even extrapolate the nonbonded forces from the current and previous forces. In comparison, our setup shown in Figure 1 is rather simple: there are no distance classes (all the nonbonded Van der Waals and Coulomb interactions are calculated together with a 5‐fs time‐step), and there is only a single step in between (when just bond, angle, and dihedral intra‐actions are calculated). For this simple case, we found that the method they named DC‐i yielded the most stable trajectories: the nonbonded forces are doubled in the even steps, and totally ignored in the odd steps in between (also called the “impulse method”^17^ or “Verlet‐I”^18^). Adding nonbonded forces every second step is still in the safe range of the impulse method, which has as advantage that it always uses exact forces that match the atom positions.

### A tuned version of LINCS to constrain bond angles

**Figure 2:**
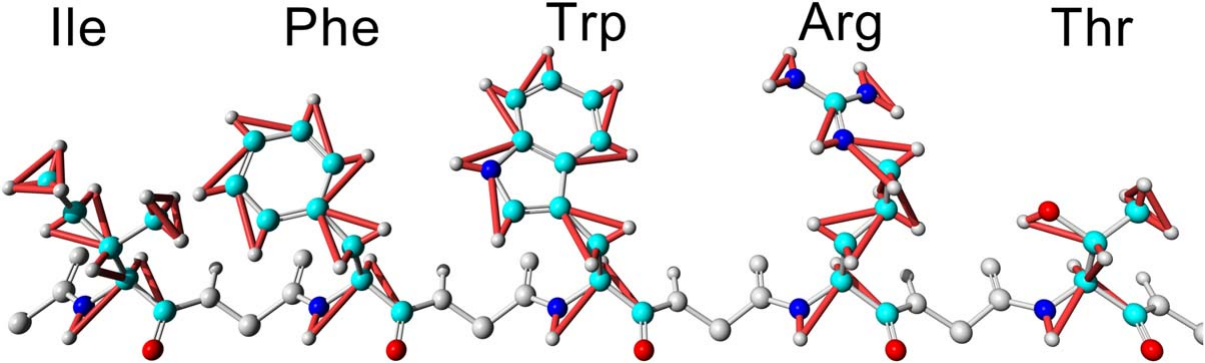
Placement of constraints to reduce the degrees of freedom of hydrogen atoms and enable larger time‐steps. The constrained distances and angles are shown for five exemplary amino acids (orange in the electronic version). The algorithm that decides which angles to constrain such that the coupling matrix can still be inverted quickly is not specific for amino acids (see Materials and Methods). [Color figure can be viewed in the online issue, which is available at wileyonlinelibrary.com.]

Figure 1 illustrates our goal to integrate bonded intra‐actions with a 2.5‐fs time‐step, and this means that vibrations of bonds and angles involving hydrogens need to be constrained. We use the very elegant LINCS algorithm,^15^ which employs a power series expansion to invert the constraint coupling matrix and to determine how to move the atoms such that all constraints are satisfied. Unfortunately, this fast approximate inversion only works as long as the simplified coupling matrix (which has zeroes along the diagonal) is sparse enough, because all absolute eigenvalues must be smaller than 1. When two constraints involve the same atom, the corresponding element in the coupling matrix becomes nonzero, so the sparsity shrinks as the connectivity between constraints grows. Consequently, the LINCS authors noted that their approach works fine for constraining bonds (also in rings), but adding angle constraints quickly raises the eigenvalues above 1, which creates a need for virtual hydrogen sites. Our alternative solution to this problem works as follows: thanks to the multiple time‐step algorithm, we can integrate the bonded intra‐actions with a 2.5‐fs time‐step (instead of the overall 5 fs time‐step). With 2.5 fs, bonds between heavy atoms are not yet critical and do not need to be constrained, which increases the sparsity of the simplified coupling matrix almost to the point where enough hydrogen angle constraints can be added to permit stable simulations (Fig. 2). Constraining all hydrogen angles is not possible, but also not necessary, as a single angle constraint per hydrogen is usually enough. We wrote “almost” and “usually,” because there is one exception: if a heavy atom has three hydrogens bound (e.g., —CH_3_, —NH_3_^+^ groups), adding constraints in a way that treats each atom equally yields a tetrahedron of six constraints (three bonds and three angles). The largest eigenvalue of the corresponding simplified coupling matrix is unfortunately 1.35. We therefore implemented a version of LINCS that handles this special case by inverting the 6 × 6 coupling matrix exactly. This requires only a few hundred CPU cycles and has no noteworthy impact on performance. A heavy atom with four hydrogens (e.g., methane) on the other hand is easy to handle again, because one can simply add two angle constraints between pairs of hydrogens (largest eigenvalue 0.82). The algorithm to decide which angles to constrain is explained in the Materials and Methods section.

One might wonder why two constraints per hydrogen are enough—after all, they cannot prevent vibrations perpendicular to the plane spanned by the constraints. The reason is that with a 2.5‐fs time‐step, not all directions are critical yet—mostly those where other hydrogens separated by four covalent bonds are close by and exert strong forces. These critical directions are protected with constraints, for example, by placing angle constraints along a chain of CH2 groups, instead of constraining just the H—C—H angle (as shown for the Arg side‐chain in Fig. 2).

Additional angle constraints generally yield larger eigenvalues, which in turn require to increase the accuracy of the LINCS algorithm to keep the constraints satisfied. Apart from trivial adjustments (like doubling the LINCS expansion order), we had to tweak the algorithm for single precision calculations. Water molecules are handled with the analytic SETTLE algorithm.^19^

A convenient aspect of our approach is that coupled constraints form small groups only. Because bonds between heavy atoms are not constrained, these groups do not extend over the entire protein, they usually do not even cross residue boundaries (Fig. 2). Consequently, special considerations regarding workload distribution (like those described for P‐LINCS^20^) are not needed when parallelizing the algorithm.

### Mixing pair list creation and force calculation

**Figure 3:**
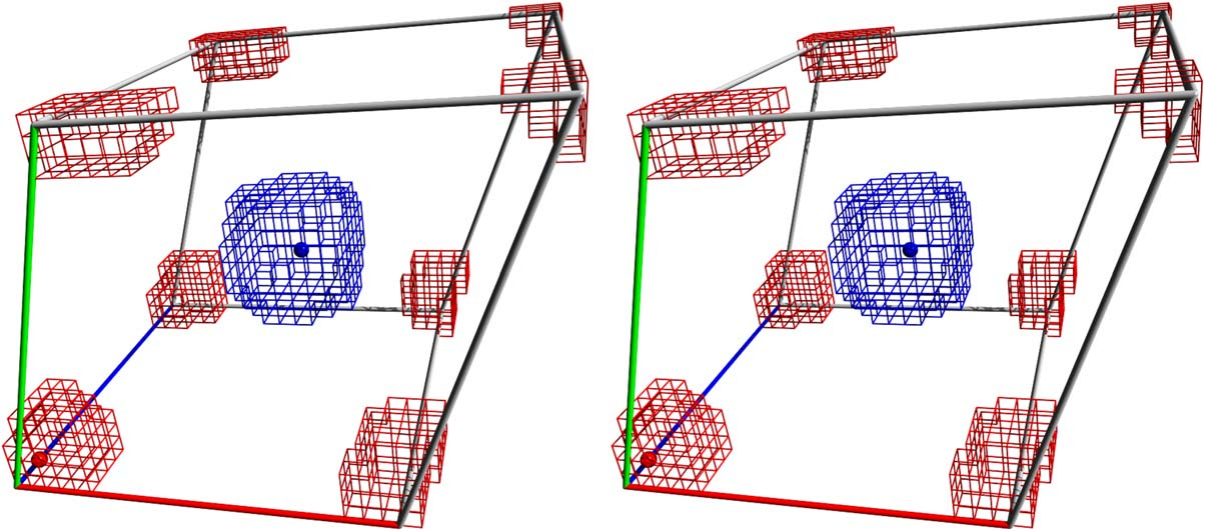
Two examples of grid‐based neighbor search in a triclinic simulation cell shown in stereo. The grid spacing is “nonbonded force cutoff/3,” so that seven cubes need to be searched along each axis to find the neighbors of the atom in the center, but four cubes can be skipped in each of the eight corners. The atom in the bottom left corner is close to three periodic cell boundaries, so cubes on the other sides must be considered too, resulting in a rather complex search pattern, which can of course be precalculated. [Color figure can be viewed in the online issue, which is available at wileyonlinelibrary.com.]

The algorithms described here have been implemented in our molecular modeling and simulation program YASARA.^21^ While there is an optional text mode interface to be run on servers, a major goal has always been to visualize the simulation on screen, allowing to dive into the system and pull atoms interactively. When we implemented this feature in 1997, CPUs were rather weak, and in order to provide a smooth interactive MD experience, YASARA did not use pair lists (i.e., arrays containing the nonbonded interaction partners for each atom). A simulation with pair lists consists of a slow step (which includes pair list creation) and a series of fast steps (using the pair lists). Such an alteration of slow and fast steps caused stutter on the screen during interactive MD runs. To ensure that each simulation step takes an equal amount of time, a grid‐based neighbor search was done at each step, intertwined with the nonbonded force calculation, so that no pair lists were needed. To maximize the performance of the grid‐search, the grid cubes should be small enough to provide a decent approximation of the cutoff sphere, and at the same time large enough to avoid useless tests of empty cubes. We obtained best performance using a grid spacing of cutoff/3 for cutoffs below 9.5 Å, and cutoff/4 above. Figure 3 shows a neighbor‐search example for an 8 Å cutoff, that is, the search space extends seven cubes along each axis. Compared to a cubic neighbor‐search volume of 7 × 7 × 7 cubes, four cubes can be skipped in each of the eight corners, reducing the search space by 10%.

Apart from the constant execution time (which was important in 1997, but has only a marginal impact on today's hardware) an algorithm that works without pair lists has two performance advantages: first, it is trivial to make the algorithm store pair lists on the fly to be used in the next steps. The resulting pair list‐based algorithm executes faster than the usual approach of first creating the pair lists and then calculating the forces, which requires to load the atom coordinates twice, possibly from slow main memory. And second, if the user wants to update the pair list at every step, one can totally skip the pair list creation.

Why would a user choose to update the pair list at every step? Apart from the interactive visualization purposes mentioned above, for example, to make sure that no interactions within the cutoff distance are missed. While the literature often mentions an idealized pair list, which is created for a somewhat larger distance **cutoff+x** and updated whenever the first atom traveled further than **x/2**, this approach is rather slow in practice, because it updates the pair list more frequently than really needed. As noted by the GROMACS team (user manual 4.6, chapter 3.4.2), it is much more efficient to just use the pair list for a certain number of steps, eventually missing some interactions within the cutoff while the atoms move. The energy drift associated with such “sloppy pair lists” can be reduced at will by increasing the pair list cutoff used during neighbor search, calculated, for example, from this empirical formula:

(1) $${\text{Cut off}}_{\text{PairList}}={\text{Cut off}}_{\text{Force}}+\frac{\sqrt{\text{Update Frequency}-1}\;\times \;\text{Time Step}\;\times \;\text{Temp}\;\times \;0.001}{M}$$

Our formula estimates how far particles with an average velocity proportional to “Temp/*M*” travel between pair list updates that are done every “UpdateFrequency” steps, given the current simulation “TimeStep.” “*M*” is the average particle mass in the simulation, water molecules are treated as single particles. We assume that particles get either closer or further away at each step, which boils down to a one‐dimensional random walk, for which the expected travel distance is proportional to the square root of the number of steps. The empirical proportionality constant 0.001 Å*Dalton/(Kelvin*fs) must be chosen as small as possible (as it increases the pair list cutoff and thus reduces the performance) and as large as necessary to reduce the energy drift to an acceptable value.

### Evaluation of simulation accuracy

Molecular dynamics simulations can be very sensitive to the protocol and algorithms used, especially when events that occur on longer time scales are investigated, like protein folding or membrane formation. Every new approximation made to gain performance must therefore be analyzed with great care. Our solution to this problem is trivial—we avoid new approximations. The methods described here either allow to calculate the same forces faster (and are thus not approximations), or recycle old approximations that have become common scientific practice. Any problems with these approaches would thus have been discovered by now. These approximations are: reducing the degrees of freedom of hydrogen atoms (e.g., with LINCS^15^ and virtual hydrogen sites^11^), sloppy pair lists (used and tested extensively in GROMACS^22^), a time‐step of 5 fs for the nonbonded interactions (the default in GROMACS when using virtual hydrogen sites), and the Verlet‐I^18^ multiple time‐step algorithm (used extensively in NAMD^14^). Most simulations were run with an 8 Å cutoff for Van der Waals‐ and direct space Coulomb forces, as this is a common choice, also when posting DHFR benchmark results (e.g., by the AMBER^23^ and OpenMM^5^ developers). This does not imply that an 8 Å cutoff is ideally suited for all purposes.^24^

The first thing to check is that the actual implementation is reliable and not suffering from problematic energy drifts, especially as it makes extensive use of single precision calculations, which are much faster on CPUs as well as GPUs (Table 1). Intuitively, one would expect the energy drift to increase with each approximation made, but this is not the case. Drifts are expressed per nanosecond and not per integration step, so if a significant part of the drift is caused by the integration procedure itself, then one can reduce the drift by increasing the time‐step (as fewer integration steps are needed per nanosecond). This allows a mixed multiple time‐step integrator with 2.5 fs (0.005 in row 4) to outperform a single time‐step integrator with 1 fs (rows 1–3). Of course, this principle no longer holds when the time‐step is increased further and starts to dominate the drift (row 5). It is also noteworthy that inaccuracies do not always cause positive drifts. Sloppy pair lists, for example, cause a negative drift, which can be adjusted at will by shifting the pair list cutoff. The resulting small negative drift in row 6 is thus due to cancelation of errors, and could be made zero or positive by increasing the 8.3 Å pair list cutoff, that is, the empirical constant “0.001” in formula 1. One should thus emphasize the importance of listing energy drifts in a step‐wise manner, while enabling the various acceleration methods, so that the real accuracy is obvious and no cancelation of errors goes unnoticed.

**Table 1:** Energy drift per nanosecond and degree of freedom during a simulation of DHFR.

Cutoff (Å)	Pair list update frequency	Interaction time step (fs)	Intra‐action time step (fs)	Constraints	Energy drift (*k*_B_ *T*/ns)
9.6	1	1.0	1.0	No	0.009
9.0	1	1.0	1.0	No	0.010
8.0	1	1.0	1.0	No	0.011
8.0	1	2.5	1.25	No	0.005
8.0	1	5	2.5	H bonds and angles	0.018
8.3/8.0	10	5	2.5	H bonds and angles	−0.006

Time‐steps for nonbonded interactions and bonded intra‐actions are listed separately. The first two rows list values in the 9 Å cutoff range to facilitate comparison with drifts reported for other MD programs.^22^ The last row uses sloppy pair lists updated every 10 steps, and a larger pair list cutoff of 8.3 Å obtained using eq. (3) with an average particle mass of 14.55 Dalton.

**Figure 4:**
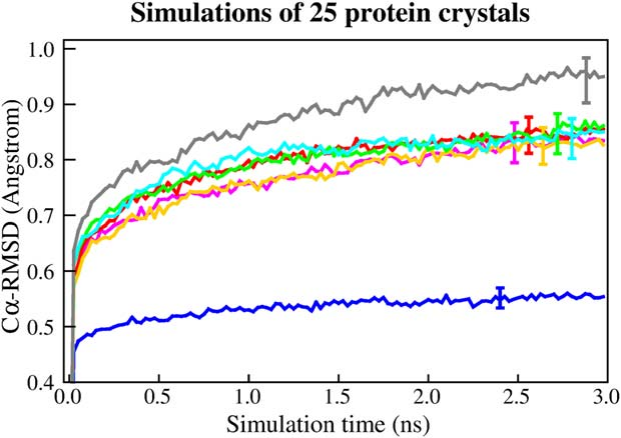
Accuracies of seven simulation protocols, measured as the average Cα RMSD of 25 protein crystal simulations with the AMBER03 force field. Error bars indicate the average and standard deviation of the RMSD during the last quarter of the simulation, averaged over the 25 simulations. The blue simulations are run at the temperature of experimental structure determination (on average 176 K), while the others are run at 298 K. The reference protocol (blue and magenta) uses PME electrostatics, a 10.5 Å VdW and direct space electrostatic cutoff, a 1‐fs time‐step, no constraints, and no pair list. The other protocols improve performance with a 8.0 Å cutoff (red), additionally a 2.5‐fs time‐step (orange), or a 5‐fs time‐step with constraints (green) and also sloppy pair lists (cyan) and have no significant impact on the RMSD, unlike a further cutoff reduction to 5.0 Å (gray). The actual performance of these protocols is shown in Figure 8.

The various approximations listed above have been described and validated in separate articles, using different methodologies. To facilitate a direct comparison, we tested each approach with an accuracy benchmark described previously^9^: simulating 25 protein crystals with the AMBER03 force field^23^ and calculating the average root‐mean‐square deviation (RMSD) from the starting structures. Using complete crystallographic unit cells ensures that all forces giving rise to the X‐ray structures are present, and RMSDs really depend on simulation accuracy and not on differences between crystal and solution environments.^9^ The reference simulation (Fig. 4, blue) was run at the temperature of the experimental structure determination (as specified in the PDB header, on average 176 K) with PME electrostatics,^25^ a 10.5 Å cutoff for Van der Waals (VdW) and direct space electrostatic forces, a single 1 fs time‐step and no constraints. Raising the temperature to the standard 298 K heavily increased the average RMSD during the last quarter of the simulation from 0.55 Å (blue) to 0.83 Å (magenta). As expected, the commonly used MD approximations had no significant impact on the RMSD: reducing the cutoff to 8.0 Å (red), additionally increasing the time‐step to 2.5 fs and using the impulse method (1.25 fs for bonded intra‐actions, orange), adding hydrogen constraints and doubling the time‐step to 5 fs (green, Fig. 1), and updating the pair list only every 10th step (cyan).

The simulations in Figure 4 are computationally expensive, because they involve 175 different trajectories, some with very slow protocols. Extending the simulation time would not improve the benchmark result, as the RMSD from the starting structures is only a useful accuracy indicator during the initial phase of a simulation—in the long term, proteins would undergo temporal partial unfolding^26^ and randomize the RMSDs. Fortunately, problems with simulation accuracy tend to show up early, as demonstrated for a cutoff reduction to 5.0 Å (gray).

### Pressure coupling without virial calculation

The most common way to run an MD simulation is the “real‐life” NPT ensemble, where the number of particles, the pressure and the temperature are kept constant. While the current temperature can simply be calculated from the atom velocities, the pressure is not trivial to handle. The most common approach is to calculate the pressure “*P*” using a formula derived from the Clausius virial theorem:

(2) $$P=\frac{2}{3\times \text{Volume}}\times \left(\text{Kinetic Energy}+\frac{1}{2}\times \sum_{i=1}^{\text{Atoms}}{\text{Position}}_{i}\times {\text{Force}}_{i}\right)$$

Unfortunately, the resulting pressures fluctuate strongly (by hundreds of atmospheres at each step), and even if the time average pressure is used to rescale the cell, one arrives at densities that are a bit off. For example, the density of water at 298 K using PME electrostatics was reported^27^ to be 0.979 g/ml instead of the expected 0.997 g/ml. Apart from changing the water model,^27^ these discrepancies can be dealt with in two ways: one favors the density and applies corrections to the pressure (e.g., to account for the truncation of attractive VdW forces at the cutoff^28^), getting closer to the right density. The other favors the pressure and argues against corrections, because they might have a negative impact (if the cutoff for VdW interactions makes waters “happy” at 0.979 g/ml, then compressing them to 0.997 g/ml could make them “feel stressed”).

**Figure 5:**
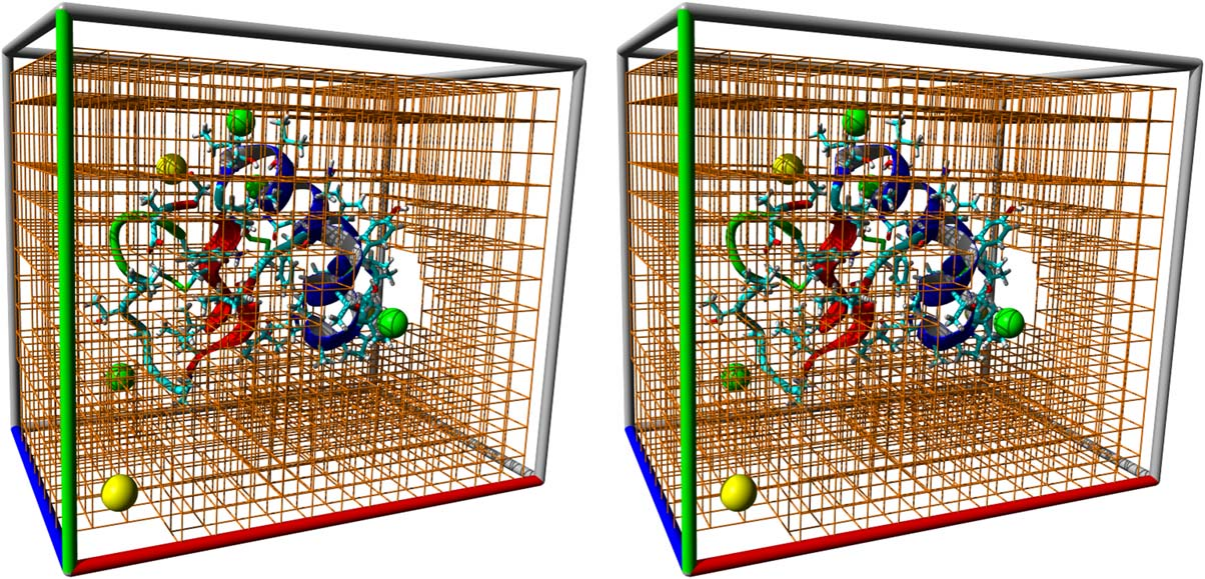
Stereo image of crambin, ions, and the grid of cubes that contain just water and can be used to quickly calculate the current water density to rescale the cell with a “densostat.” The grid was obtained by excluding the 3 × 3 × 3 = 27 cubes around each nonwater atom, which mostly covers the first hydration shell. The ions shown correspond to a physiological NaCl concentration of 0.9% (154 mM). At this concentration, ∼78% of the solvent cubes can be included in the density calculation. The fraction of useful cubes drops slowly with increasing NaCl concentration, reaching 50% at about 4.5% NaCl (770 mM). We also tested the exclusion of larger parts of the hydration shells (57 cubes around each nonwater atom, that is, the big cube of 3 × 3 × 3 cubes above, plus a cross of five cubes on each of its six sides), and found that the accuracy of the density calculation did not improve significantly, while considerably fewer cubes could be used (70% at 0.9% NaCl, reaching 50% already at ∼2.3% NaCl). In case of very high salt concentrations or mixed solvents, where not enough water cubes are available, one can of course run a reference simulation with a barostat to measure the density of the entire solvent and plug that value into the densostat, which is then based on solvent cubes instead of water cubes. [Color figure can be viewed in the online issue, which is available at wileyonlinelibrary.com.]

Our approach does not choose a side, but lets the user decide. It is based on an assumption that is implicit in all pressure coupling protocols with cell rescaling—that pressure is not a localized property, but spreads through the cell. The pressure in the left half of the cell is the same as in the right half, the pressure in the solute is the same as in the solvent. This implies that one only needs to know the solvent pressure, which allows to take the shortcut shown in Figure 5. Having placed a grid in the simulation cell, all grid cubes that contain just water atoms (and have 26 neighbors with just water atoms) are tagged. Then, the masses of the water atoms in the tagged cubes are summed and divided by the volume to obtain the current water density. The grid is simply the neighbor search grid with ∼2.6 Å spacing (Fig. 3). Contrary to the virial, the water density shows only small variations (whenever atoms change a cube), so that it is enough to calculate it every 10 steps and then average 50 measurements to obtain a stable result that can be plugged into a “densostat” (similar to a Berendsen barostat^29^), which defines a scaling factor “*S*” for the atom coordinates to reach the desired water density:

(3) $$S=\max\left(0.999,\min\left(1.001,\sqrt[{3}]{1+C\times \left(\frac{{\text{Density}}_{\text{measured}}}{{\text{Density}}_{\text{set}}}-1\right)}\right)\right)$$

The “max” and “min” functions make sure that the cell is never scaled by more than 0.1%, even if the user chooses a large coupling strength “*C*” or if the density difference is large (we found 0.1% to be a reasonable limit to avoid a temperature rise caused by scaled bond lengths). The time average density is used so that fluctuations are not artificially suppressed (which can be a problem with weak coupling methods, especially if the simulated system is small^30^).

Our “densostat” has two advantages: first, it lets simulations run about 8% faster. The reason is that the virial calculation—even though it looks fast and simple in eq. (1)—requires special care when handling forces that cross periodic boundaries, which effectively pulls it into the inner loops of the force calculation. And second, the densostat makes it trivial to reach exactly the right density (if that is desired). Those who prefer the right pressure can simply run a reference simulation of water with a barostat, and their favorite cutoff and temperature, and use the resulting water density as the densostat target value.

The disadvantage is obvious: as the density is a scalar, the densostat fails when the pressure cannot be expressed as a scalar, that is, when the three values along the trace of the pressure tensor deviate from each other. This happens when the solute spans the entire cell, so that solvent molecules cannot travel freely to spread the pressure uniformly. The most common examples are proteins embedded in a membrane or protein crystals. These need to be handled the classic “virial” way, using different scaling factors for each cell axis. We do not claim that the densostat can replace the virial calculation in all the other applications of molecular dynamics simulations, but we found no influence on the dynamics of the simulated system. Table 2 shows an analysis of atomic B‐factors extracted from 150 ns simulations of DHFR in solution at 298 K (using the cyan protocol in Fig. 4). With a barostat (Materials and Methods), the average heavy atom B‐factor was 78 Å^2^. Running the same 150 ns simulation a second time, but with a different random number seed for the initial velocities, yielded heavy atom B‐factors that differed on average by −18 ± 59 Å^2^. With the densostat, B‐factors differed by 5 ± 48 Å^2^, so the densostat had no larger impact than the random number seed. However, after changing the force field from AMBER03 to AMBER99,^31^ B‐factors differed by 26 ± 58 Å^2^. Also, the cell volume during the last 75% of the simulation was not significantly different.

**Table 2:** Influence of force field, pressure coupling method, and random number seed on the B‐factors and cell volume extracted from 150‐ns simulations of DHFR in solution at 298 K (at room temperature and without crystal packing, B‐factors are higher than those found in X‐ray structures).

Force field	Pressure coupling	Random seed	Average B‐factor (Å^2^)	Average B‐factor difference from first row (Å^2^)	Cell volume (Å^3^) with 23,786 atoms
AMBER03	Barostat	0	78	0 ± 0	240,645 ± 218
AMBER03	Barostat	1	60	−18 ± 59	240,700 ± 220
AMBER03	Densostat	0	83	5 ± 48	240,560 ± 342
AMBER99	Barostat	0	104	26 ± 58	240,744 ± 223

These data reflect the common observation that simulation time‐scales of proteins are usually too short to reach exhaustive sampling, so that the results often depend considerably on the initial conditions. This dependence can easily mask variations in the simulation protocol, like the switch between barostat and densostat shown in Table 2.

To verify that the densostat also works for small systems, which can be sampled well and thus show a stronger dependence on the simulation protocol, we ran microsecond simulations of a well known model system—the alanine dipeptide—and extracted the free energy landscape.

**Figure 6:**
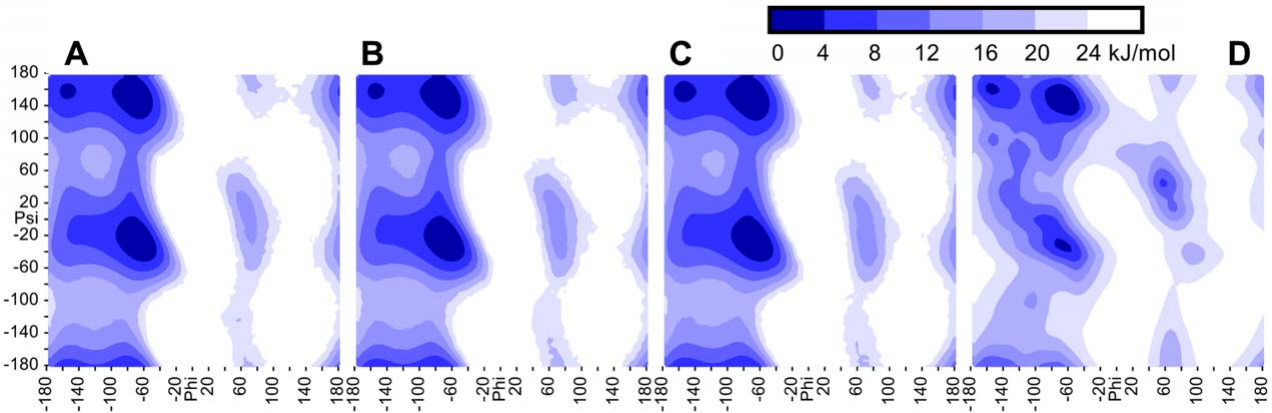
The free energy landscape of the alanine dipeptide, showing the *φ*/*ψ* map of the first alanine. (A) Microsecond simulation at 298 K with a conservative protocol (single 1 fs time‐step, exact pair list updated each step, no constraints, barostat with 1 bar). (B) The same simulation again, but with different random seed and different initial conformation of the peptide. (C) Simulation with our fast protocol (multiple 5 fs time‐step, sloppy pair list updated every 10 steps, bond and angle constraints, densostat with 0.97 g/ml water density^27^). (D) For comparison, the corresponding *φ*/*ψ* map of alanine, extracted from high‐resolution X‐ray structures in the PDB, with an additional round of smoothing to provide knowledge‐based torsion forces in the YASARA force field.^6^ [Color figure can be viewed in the online issue, which is available at wileyonlinelibrary.com.]

Early simulations of the alanine dipeptide lasted a few nanoseconds and required tricks to enhance sampling, like thermodynamic integration of perturbed trajectories.^32^ Fortunately, the microsecond simulations possible on today's hardware provide sufficient sampling—the plot of the *φ*/*ψ* free energy map shows only marginal dependence on the initial conditions (Figs. 6A and 6B). Enabling the fast simulation methods described here (including the densostat) yields comparable differences (Fig. 6C). In contrast, Figure 6D shows the free energy map of alanine, extracted from high‐resolution X‐ray structures in the PDB. Differences are of course expected, but we note that the positions of the energy minima match well.

### Multithreaded force calculation methods and overall performance

**Figure 7:**
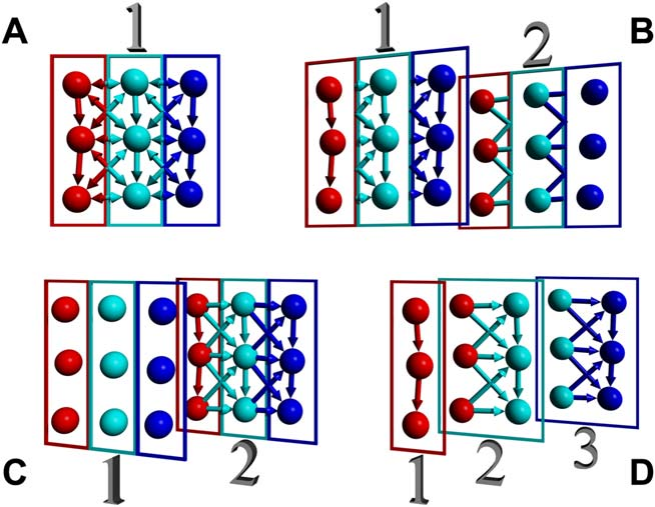
Four methods to parallelize nonbonded force calculation. In this example, three threads (red, cyan, and blue) calculate the forces acting on nine atoms. Each arrow corresponds to one interaction within the cutoff range (which includes direct and diagonal neighbors). The arrow color shows which thread calculates the force, which is added to the atom pointed at. If the arrow has a single head, the force is also subtracted from the other atom (and thus needs to be calculated once only). If the arrow has two heads, the same force needs to be calculated twice (by two threads), which reduces performance. The colors of the rectangles indicate which thread owns the force table(s). (A) Single force table, forces that cross thread boundaries need to be calculated twice. (B) Two force tables with atomic subtractions of forces that cross thread boundaries in Table 2. (C) Two force tables using transactional memory, the first table is only needed if a transaction fails. (D) One separate force table per thread, which need to be summed in the end.

We evaluated the performance of the various methods by running the well‐known DHFR benchmark, a simulation of dihydrofolate reductase (3158 protein atoms and 20,628 water atoms) with PME electrostatics.^25^ Today's CPUs can execute a steadily growing number of parallel threads, so performance depends to a large extent on the way the nonbonded force calculation is distributed. We analyzed four different approaches, all of which distribute atoms among threads by dividing the cell into “thread regions,” from left to right. Atoms are reordered such that those in the same region are stored next to each other in memory (which optimizes cache usage and nonunified memory access). The easiest approach is shown in Figure 7A. It is based on a single table of atomic force vectors, one for each atom, which is shared among the threads. Each thread calculates and adds the forces acting on its atoms. If an interacting atom belongs to the same thread, the force is immediately subtracted (i.e., added in reverse direction) there too. Otherwise the force needs to be calculated twice (once by each thread), because one thread cannot simply change forces belonging to other threads (which would cause a “race condition”).

**Figure 8:**
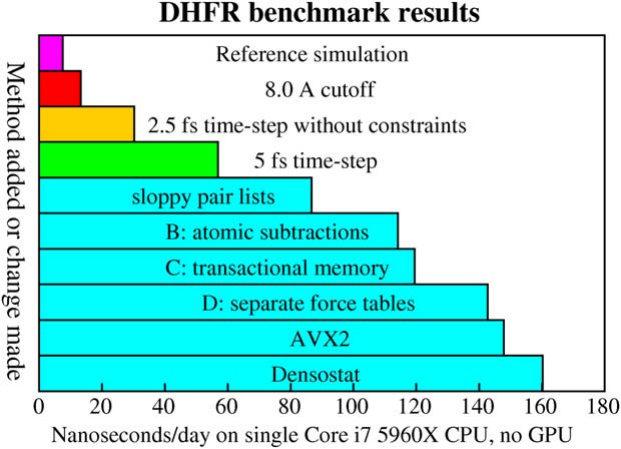
Performance of 10 simulation protocols in the DHFR benchmark. The reference protocol (top) uses a 10.5 Å cutoff, a 1‐fs time‐step, no constraints, no pair list, a barostat, and a single force table shared among threads (Fig. 7A). [Color figure can be viewed in the online issue, which is available at wileyonlinelibrary.com.]

With the most conservative simulation parameters (10.5 Å VdW and direct space electrostatic cutoff, 1 fs time‐step), using the AVX instruction set and a classic virial‐based barostat, method A yields 7.5 ns/day on an Intel Core i7 5960X CPU with 16 threads and 3.6 GHz (Fig. 8, magenta). If the cutoff is reduced to 8.0 Å, performance increases to 13.3 ns/day (red). Activating the mixed multiple time‐step without constraints (2.5 fs) yields 30.2 ns/day (127% more, orange). Constraints (5 fs time‐step) almost double the speed (56.9 ns/day, green), and sloppy pair lists add 52% to 86.7 ns/day (cyan).

Method B (Fig. 7B) uses two force tables. Forces are always calculated once and added in force table 1. If the interacting atom belongs to the same thread, the force is also subtracted in force table 1, otherwise it is atomically subtracted in force table 2. “Atomic” means that the subtraction cannot be interrupted by another thread, avoiding race conditions. This is achieved by acquiring a simple spinlock (using the x86 instruction sequence “loop: xor eax,eax/pause/lock cmpxchg/jnz loop”), performing the force subtraction, and releasing the lock. We use one lock per atom, which is placed as the fourth element of the force vector (a force vector normally needs 3 × 4 bytes storage, but SSE requires 16‐byte alignment, so a fourth element is included for padding). Both tables need to be added in the end. Method B increases performance by 32% to 114.1 ns/day.

Method C (Fig. 7C) also uses two force tables and employs the new transactional memory extension TSX, introduced by Intel with the Haswell CPU architecure. TSX provides an instruction “xbegin,” which starts a memory transaction. Right afterward, the program is allowed to behave as if no other threads were present, adding and subtracting forces in table 2 at will. When done, the program issues the “xend” instruction, which tells the CPU to commit the transaction. If another thread just by chance tries to access the same force vectors at the same time, the transaction fails, the CPU restores the state before the transaction and executes a fallback path instead, which is simply method B. The fraction of aborted transactions depends on the number of atoms and the number of threads working on the atoms. When simulating DHFR with eight threads, 5% of the transactions fail. With increasing system size, the failure rate approaches 2%. As TSX has a considerable overhead, it is not trivial to outperform method B. Intel recommends that a transaction should last about 400 ns, we got good results by bundling 8 to 16 force additions (and the corresponding subtractions) in a single transaction. This yields an improvement of 5% (measured on a Core i7 4770, as Intel unfortunately disabled TSX in the Core i7 5960X due to a hardware issue).

Method D (Fig. 7D) uses one force table per thread. Each thread can thus add and subtract forces without any danger of collisions. The drawback is that this approach requires more memory to store forces, and the forces for each atom have to be summed in the end. The good news is that—if the system is large compared to the number of threads—a certain atom is usually only touched by 2–4 threads, so that only 2–4 forces per atom need to be stored and summed. Method D performs best, improving simulation times by 19% to 142.7 ns/day.

Methods A and D have the inherent advantage of being reproducible, so that one can obtain the very same MD trajectory twice in a row. Methods B and C add forces in random order, and as A+B+C does not exactly equal A+C+B in floating point operations, they yield marginally different forces, which quickly causes trajectories to diverge.^33^

Only time will tell which of the four methods wins on future CPU generations. Four‐socket systems with Haswell Xeon CPUs may support up to 144 threads, requiring method D to store and sum so many forces per atom, that method C could run faster. Intel's new Xeon Phi “Knights Landing” should arrive in 2015 with 288 threads and no TSX, which could make method A or B win.

**Figure 9:**
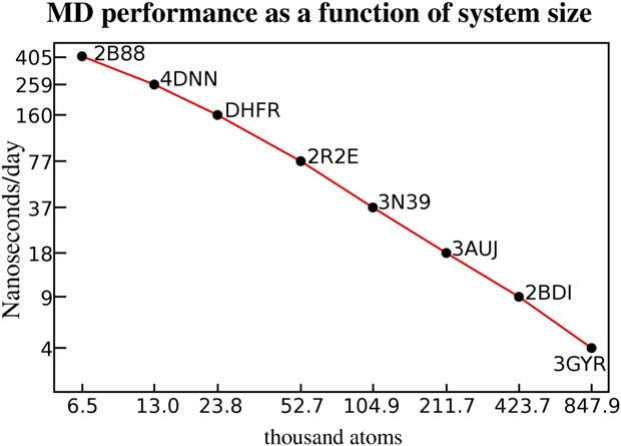
Molecular dynamics performance on a Core i7 5960X CPU for eight proteins, their PDB IDs are shown. The simulation protocol is the last (fastest) from Figure 8. Both axes use logarithmic scaling, the system size approximately doubles each step. While the structures in the middle show a linear O(N) scaling, the small structures 2B88 and 4DNN do not perform ideally, because the cost to launch and synchronize 16 threads starts to dominate the computation. For very large structures like 3GYR, the performance is reduced because the fast Fourier transform of the PME calculation has only O(N × log N) scaling and becomes the bottleneck. [Color figure can be viewed in the online issue, which is available at wileyonlinelibrary.com.]

The AVX2 instruction set released by Intel in 2013 helps a bit (4% to 147.8 ns/day), thanks to the doubled register space for integers, which is needed when calculating force‐table lookup indices. The densostat finally adds 8% to 160.2 ns/day. The performance of this protocol for a wide range of system sizes is shown in Figure 9.

The benchmark results shown in Figures 8 and 9 have been obtained with the 32bit version of YASARA, because the 64bit version was not completely finished at the time of writing. As 64bit operating systems happily run 32bit software, this is not an issue in practice. 64bit mode offers twice as many CPU registers, which could boost performance beyond 200 ns/day (estimated from the observation that the GROMACS 64bit version runs about 30% faster than the 32bit version).

## Materials and Methods

### Choice of programming language

To exploit the full potential of today's CPUs, one needs to make extensive use of the various vector instruction sets (e.g., SSE and AVX), where a single instruction operates on multiple data elements in parallel (SIMD approach). Although compilers could in theory do that automatically, it does not work well enough in practice. Instead, the developer must write code that explicitly uses these instruction sets, either by programming directly in assembly language, or by using “intrinsics,” small C/C++ functions that operate on vector data types and map almost directly to the corresponding assembly instructions, so that the compiler has an easy job. Both approaches have disadvantages: assembly language is hard to maintain (especially with respect to local variables and register spilling), while intrinsics are rather cryptic and require to disassemble the code to check that the compiler really did what it was supposed to (which is very difficult for large functions), and both suffer from the major problem that one needs to rewrite or at least adapt the code for each SIMD instruction set (and almost every new CPU comes with additional SIMD instructions). For a general molecular modeling application like YASARA, which uses high‐performance code throughout (including molecular graphics), both approaches are impractical.

Our solution to this problem was to develop a “meta assembly language” named PVL (portable vector language), which supports all the low‐level performance tricks possible in assembly, but keeps the administration of the code nevertheless simple. As PVL is not publicly available, we briefly describe the main features to help reproducing the results: PVL hides the complexity of the various SIMD instruction sets by providing its own simple vector data types and instructions. As a result, one needs to write the code only once, and PVL translates it to currently 16 different versions (SSE, SSE2, SSE3, SSSE3, SSE4, AVX, AVX+FMA3, AVX2, each for 32 and 64bit mode. Support for various 3DNow! combinations, for example, SSE2+3DNow!, was dropped only recently, when AMD discontinued the latter). The different code paths can be packed into the same executable and chosen at run‐time, so that a single executable runs optimally on all CPU architectures (intrinsics normally require to provide a separate executable for each CPU type). PVL can create multiple similar functions from a single parent (e.g., to calculate nonbonded forces with and without the virial, with PME or with a switching function etc.). PVL allows to define vectors with variable length to optimally fill the available registers (AVX registers can store eight floats, while SSE registers can only store 4, and 64bit mode has twice as many registers as 32bit mode). For example, nonbonded forces are calculated for 8 atoms in parallel in 32bit SSE code, for 16 atoms in 32bit AVX code, and for 32 atoms in 64bit AVX code. PVL takes care of local variables and function parameters, addressing them via the stack pointer, so that no frame pointer is needed (reducing register shortage in 32bit mode). PVL supports position independent code (needed on Android), automatic register spilling, loop unrolling, and nested functions: when using SIMD instructions, loop unrolling is not optional but obligatory (because it is very slow to access the *i*th element of a vector register if “*i*” is a variable). This quickly blows up the code beyond the instruction cache size (e.g., for a nonbonded force kernel, PVL creates 50,000 lines of assembly code, taking ∼115 kb memory). Consequently, in‐lining functions is a hopeless task, and one needs a way to quickly call functions without overhead. PVL allows to embed a function inside another function, so that the callee can access all the local variables and function parameters of the caller without having to pass them explicitly.

### Choice of data structure layout

Programming for SIMD architectures involves the difficult choice between two competing approaches to arrange the data in memory: structures of arrays and arrays of structures. The first places all data of the same type next to each other (e.g., all atom X‐coordinates), so that loading a SIMD register from memory fills it with data of the same type (e.g., one AVX register with the X‐coordinates of eight atoms, one register with the Y‐coordinates etc.). Operating on these data is then trivial, for example, one would perform three multiplications and two additions to calculate eight dot products in parallel. The second approach places all data belonging together next to each other (e.g., an atom's X, Y, Z coordinates and charge), so that loading a SIMD register from memory fills it with the data of one or more atoms (e.g., one AVX register with the positions and charges of two atoms). The second approach is far less convenient, because it requires cumbersome shuffling and “horizontal operations” (like adding two neighbor values in the same SIMD register), for example, one would perform one multiplication and two horizontal additions to calculate just two dot products in parallel. Nevertheless, we chose this approach in our MD algorithms, for three good reasons: first, it improves memory locality and thus cache hit rate (the position of an atom can be loaded with a single instruction from the same cache line and does not have to be gathered from three different locations). Second, current SIMD instruction sets provide good support for the difficult horizontal operations within a register, for example, the AVX “vdpps” instruction calculates two dot products in one shot. And third, it requires far fewer registers, which avoids expensive register spilling to memory. For example, to store the position of a water molecule, the “structures of arrays” approach needs nine AVX registers (to store the X/Y/Z coordinates of one O and two Hs), while the “array of structures” approach only needs 1.5 (half a register for the oxygen position, and one register for the two hydrogen positions). As the CPU only has 8 registers in 32bit and 16 registers in 64bit mode, this helps a lot.

### Force interpolation

Force calculation in accurate and fast MD simulations involves interpolation from lookup tables, because the treatment of longrange electrostatics with PME requires to evaluate the Gauss error function to determine the real space damping factor,^25^ which cannot be done fast enough. As lookup tables “pollute” the cache and induce slow main memory accesses, our implementation uses only four: one table with the general PME damping factors as a function of distance, and three tables with O—O, O—H, and H—H forces between water molecules. Lennard Jones forces involving solute atoms are thus calculated explicitly. We use linear interpolation, which has become common practice in many popular MD programs thanks to GPUs and their built‐in linear interpolation hardware (normally used for texture mapping). As described in detail previously,^4^ the linear interpolation error is about 1e^−6^. This matches the difference one gets when summing up the forces acting on an atom in a different order using single precision floats (which only have ∼7 significant digits). The interpolation is performed using the AVX2 “vcvtps2dq” instruction to convert the distance to an integer index for the look‐up table, two “vgatherdps” instructions to fetch the two boundary values, “vpand” and “vcvtdq2ps” to calculate the two scaling factors, and one multiplication combined with a fused multiply‐add to calculate the result. The drawback of “vgatherdps” is that it blocks three of the eight AVX registers and runs only marginally faster than manual gathering.

### Algorithm used to constrain distances and angles

All bonds and selected angles involving hydrogens are constrained with a tuned variant of the LINCS method. “Constraining the bond angle A‐B‐C” means that the distance between atoms A and C is constrained to sqrt(sqr(AB)+sqr(BC)−2*AB*BC*cos(ABC)), where AB, BC, and ABC are the equilibrium distances and angle assigned by the force field. The tuning involves optimization for single precision calculations (next paragraph) and the handling of heavy atoms with three bound hydrogens (e.g., the CH_3_ groups in Fig. 2). In this case, the six constraints (three bonds and three angles) form a tetrahedron, and the largest absolute eigenvalue of the simplified coupling matrix **A** is 1.35, so that the approximate LINCS matrix inversion (**1**‐**A**)^−1^ = **1+A+A** ^2^+**A** ^3^+. fails. We therefore invert the 6 × 6 matrix **1‐A** exactly, noting that the same inverse can be used in both LINCS steps (the initial projection and the correction for rotational lengthening). We do not take advantage of the fact that **1‐A** is symmetric, contains a few zeroes and only ones along the diagonal, but instead simply use the fastest of Intel's SSE‐optimized 6 × 6 matrix inversion routines (document AP‐929, order number 245044‐001).

To apply the constraints with sufficient accuracy (i.e., yielding a sufficiently small energy drift), we use a LINCS matrix expansion order of 8 and perform the correction for rotational lengthening three times in a row. The LINCS algorithm originally described^15^ takes as input the old and new atom coordinates (obtained from the MD integrator) and then iteratively adjusts the new coordinates until the constraints are satisfied. Unfortunately, single precision floating point numbers are a troublesome but unavoidable way of storing absolute atom coordinates, which get less accurate the further they are away from the origin. Every coordinate change is thus coupled with a loss of accuracy and should be avoided, which makes the many LINCS iterations required to handle angle constraints problematic. Relative coordinates on the other hand make optimal use of the 32 bits available. We therefore changed the MD integrator to provide LINCS with the old positions and the steps to the new positions instead. The steps are then adjusted by LINCS, and only added to the old positions in the end, yielding more accurate results and smaller drifts.

### Algorithm used to select constrained angles

The angles to constrain must be chosen carefully so that the eigenvalues of the simplified constraint coupling matrix stay below 1. We use a recursive algorithm, which is centered on the function FixHydrogenAngles, whose pseudocode is given below:[Link]

FixHydrogenAngles(done[],atm,lastatm)
{// 'done' is a table that flags atoms which have already been analyzed.
// The analysis starts at 'atm' and should not recurse to 'lastatm'.
// First make sure that each atom is analyzed only once:
if (done[atm]) return
done[atm]=1
// Maybe we will recurse to 'nextatm', but not yet
nextatm=NONE
// Store the bound hydrogens in 'hydtab' and their number in 'hydrogens'
GetBoundHydrogens(hydtab,&hydrogens,atm)
if (hydrogens==4)
{// Handle four hydrogens like methane with two constraints
FixAngle(hydtab[0],atm,hydtab[1])
FixAngle(hydtab[2],atm,hydtab[3])}
if (hydrogens==3)
{// Handle three hydrogens like CH3,NH3 groups with three constraints
FixAngle(hydtab[0],atm,hydtab[1])
FixAngle(hydtab[1],atm,hydtab[2])
FixAngle(hydtab[2],atm,hydtab[0])
// Don't recurse if the heavy atom bound to atm is sp3 with <=1 hydrogens
nextatm=BoundHeavyAtom(atm)
if (nextatm!=NONE and BoundHydrogens(nextatm) <=1 and Bonds(nextatm)>3) nextatm=NONE}
if (hydrogens==2)
{if (Bonds(atm)>3)
{// Two hydrogens bound to sp3 atom. These need constraints to the next heavy atom:
nextatm = A heavy atom bound to atm, which is not lastatm, which has <3 hydrogens,
which does not already have an angle constraint atm‐nextatm‐x,
and which has the highest score. The score is 3 if nextatm has two bound
hydrogens (it's best if we continue along a ‐CH2‐ chain), 2 if it has 0
hydrogens (but also OK to end at an atom without hydrogens), 1 if it has
1 hydrogen and 3 bonds, otherwise 0.}
if (nextatm!=NONE)
{// Found a bound heavy atom that can take the constraints
FixAngle(hydtab[0],atm,nextatm)
FixAngle(hydtab[1],atm,nextatm)
// Again, don't recurse if nextatm is sp3 with <=1 hydrogens, these are handled later
if (BoundHydrogens(nextatm)<=1 and Bonds(nextatm)>3) nextatm=NONE}
else
{// Two hydrogens bound to sp2 atom (or no nextatm found), these can safely be coupled
FixAngle(hydtab[0],atm,hydtab[1])}}
if (hydrogens==1)
{// A single hydrogen gets a single constraint. Find best partner atom.
nextatm = A heavy atom bound to atm, which has <3 hydrogens and the best score.
The score is ‐(number of bound hydrogens + number of angle constraints
nextatm‐x‐y)*4. If nextatm==lastatm or there already is an angle constraint
atm‐nextatm‐x, then score=score‐20. If nextatm is not in the same residue
as atm, then score=score‐10.
if (nextatm!=NONE) FixAngle(hydtab[0],atm,nextatm)}
// Recurse if it makes sense
if (nextatm!=NONE) FixHydAngles(done,nextatm,atm)}



The function FixHydrogenAngles is called first for all atoms with three bound hydrogens (—CH3, —NH3^+^), second for all atoms with one hydrogen and two bonds (—OH, —SH), third for all atoms with two hydrogens that have at least one atom with a single and at most one atom with two hydrogens bound (this traverses along —CH2— chains, leaving maximum options for the atoms with a single hydrogen), fourth for all atoms with two hydrogens that have at most one atom with two hydrogens bound (this traverses along the remaining —CH2— chains), fifth for all atoms with a single hydrogen that have at most one atom with a single hydrogen bound (this traverses along —XH— chains in rings), and finally for all remaining atoms.

The above heuristic recipe was tuned by analyzing a large number of organic molecules, and was the easiest approach that gave optimum results (i.e., the largest number of constraints below the eigenvalue limit 1) without resorting to a global optimizer, which would have raised the complexity of the approach.

### Alanine dipeptide simulations

The alanine dipeptide was built with YASARA,^21^ adding acetyl‐ and *N*‐methyl capping groups. The system consisted of ∼3000 atoms (32 peptide atoms, 981 water molecules, and three ion pairs, that is, 0.98% NaCl). The force field was AMBER03, simulations were run at 298 K with the protocols described in the caption of Figure 6. After an equilibration period of 1 ns, the current *φ*/*ψ* dihedrals were calculated every 50 fs and mapped to a two‐dimensional grid with a resolution of 5° (72 × 72 bins), then the corresponding counter was incremented. After a microsecond, the probability in each grid bin was obtained by dividing with the total number of counts, converted to a free energy using the well known Boltzmann formula Energy = −BoltzmannConstant × 298 × ln(Probability), shifted so that the energy minimum was at 0, and visualized using the marching squares algorithm for seven contour levels with a spacing of 4 kJ/mol. The YASARA macro used to perform these tasks can be found in the documentation of the free YASARA View program version 15 or later, at Commands > Options > Tables > Tabulate.

### DHFR benchmark details

All dihydrofolate reductase benchmark results were obtained by compiling and running on an Intel Core i7 5960X CPU with 3.6 GHz, the latest RedHat Enterprise Linux 7 (free CentOS version) and GCC 4.8. Turbo boost (Intel's dynamic overclocking feature) was disabled to ensure consistent timings. Hyperthreading was enabled, so that 16 threads were available. PME electrostatics were calculated with a grid spacing < 1 Å and fourth‐order B‐splines. Pressure coupling was done as indicated, either based on the density (see Results section), or on the pressure calculated from the virial. In the latter case, the Berendsen barostat^29^ was fed with the time average pressure to avoid the suppression of fluctuations, which have been analyzed in detail for the weak coupling methods.^30^

## Footnotes

You can view this pseudocode in the following link www.yasara.org/constraints.
